# A catalog of potential putative functional variants in psoriasis genome-wide association regions

**DOI:** 10.1371/journal.pone.0196635

**Published:** 2018-05-01

**Authors:** Yan Lin, Lu Liu, Yujun Sheng, Changbing Shen, Xiaodong Zheng, Fusheng Zhou, Sen Yang, Xianyong Yin, Xuejun Zhang

**Affiliations:** 1 Institute of Dermatology, Department of Dermatology, The First Affiliated Hospital, Anhui Medical University, Hefei, Anhui, China; 2 Department of Dermatology, The Fourth Affiliated Hospital, Anhui Medical University, Hefei, Anhui, China; 3 Key lab of Dermatology, Ministry of Education, Anhui Medical University and State Key lab of Dermatology Incubation, Hefei, Anhui, China; 4 Department of Dermatology, China-Japan Friendship Hospital, Beijing, China; 5 Center for Statistical Genetics, Department of Biostatistics, University of Michigan, Ann Arbor, Michigan, United States of America; Kunming Institute of Zoology, Chinese Academy of Sciences, CHINA

## Abstract

Psoriasis is a common inflammatory skin disease, with considerable genetic contribution. Genome-wide association studies have successfully identified a number of genomic regions for the risk of psoriasis. However, it is challenging to pinpoint the functional causal variants and then further decipher the genetic mechanisms underlying each region. In order to prioritize potential functional causal variants within psoriasis susceptibility regions, we integrated the genetic association findings and functional genomic data publicly available, i.e. histone modifications in relevant immune cells. We characterized a pervasive enrichment pattern of psoriasis variants in five core histone marks across immune cells/tissues. We discovered that genetic alleles within psoriasis association regions might influence gene expression levels through significantly affecting the binding affinities of 17 transcription factors. We established a catalog of 654 potential functional causal variants for psoriasis and suggested that they significantly overlapped with causal variants for autoimmune diseases. We identified potential causal variant rs79824801 overlay with the peaks of five histone marks in primary CD4+ T cells. Its alternative allele affected the binding affinity of transcription factor *IKZF1*. This study highlights the complex genetic architecture and complicated mechanisms for psoriasis. The findings will inform the functional experiment design for psoriasis.

## Introduction

Psoriasis is a common chronic immune-mediated inflammatory skin disease, well characterized by abnormal hyperproliferation and differentiation of keratinocytes, and infiltration of T lymphocytes in the psoriatic lesion [1, 2]. Despite the mechanism is unclear, the incidence of psoriasis is widely recognized to be attributed to genetic variants and environmental factors [2]. Recently, the understanding for genetic contribution to the risk of psoriasis has been dramatically improved through genome-wide association studies (GWASs) in multiple ethnic populations, which have totally identified less than 100 psoriasis susceptible regions [3–16]. The GWAS findings are typically single nucleotide polymorphisms (SNPs, termed as “tagSNPs”). The majority of them act as proxies for causal variants through linkage disequilibrium (LD) [17]. It is crucial to identify the causal variant within each region in order to decipher disease mechanism in psoriasis [18].

However, the complex genetic architecture of psoriasis poses a great challenge [19]. Firstly, the causal variants are usually highly correlated with tagSNPs; however, there are normally hundreds and even thousands of such variants linking with tagSNPs in each region. As a result, it is hard to distinguish the real one for the complex LD structure. Secondly, the majority of GWAS implicated SNPs are non-coding genome sequences, which in the past have been considered to be the unexplored territory. It remains challenging to interpret its biological consequence [20]. Nevertheless, it is widely believed that a true causal variant affects disease biological pathophysiology through changing the activities and function of specific cell types and/or tissues by complicated biological pathways [21]. To interrogate the disease mechanism underlying each causal variant, it is critical to use the most biological relevant cells or tissues.

Nowadays, more and more high throughout functional genomic data for hundreds of cells or cell lines has become publicly available, especially the data from the Encyclopedia of DNA Elements (ENCODE) Consortium and RoadMap epigenetics Project [22, 23]. The ENCODE Consortium has defined hundreds of thousands of cell-type-specific distal regions and demonstrated that the non-coding genome contained well-demarcated gene regulatory regions, for example gene enhancer, promoter, transcription factor binding sites (TFBS) [22, 24], in some of which the GWAS findings clearly and significantly enrich for common diseases/complex traits [19, 20, 25, 26]. The functional genomic data provides a great opportunity to interpret known disease GWAS findings. The integrative analysis of GWAS evidences and functional genomic data in the disease-specific cells/tissues would help prioritize the causal variants in GWAS regions. In 2013, Rhie SK et al. interrogated 71 risk loci in functional genomic data for human mammary epithelial cells (HMEC) and three other cell lines, and concluded 1,005 potential functional SNPs for breast cancer [27]. In 2014, 51 variants were implicated within gene promoters and enhancers for colorectal cancer by using gene expression data from normal and tumor cells. Especially, the regulatory effect of one implicated functional enhancer variant was then demonstrated through clustered regularly interspaced short palindromic repeats (CRISPR) nucleases technology [21].

To this end, we hypothesized that to use the functional genomic data in biologically relevant immune cells/tissues would narrow down the functional variants and elucidate the biological mechanisms in psoriasis GWAS regions. In the present study, we characterized the enrichment of psoriasis SNPs in chromatin states across immune cells/tissues, and then integrated the psoriasis SNPs and histone marks peaks in relevant CD4+ T cells. We concluded a catalog of potential putative functional variants (PFV) for psoriasis. The findings shed light on the disease mechanisms and will inform the designs of functional experiments for psoriasis.

## Materials and methods

### Psoriasis GWAS variants

We identified 75 genetic variants (hereafter “tagSNPs”) through an extensive manual literature review, which have been established by March 2015 for the risk of psoriasis with genome-wide significant evidence in GWASs of Eastern Asian and European populations. The full list of tagSNPs is available in S1 Table.

### Enrichment of histone marks and transcription factors

To evaluate and characterize the pattern of psoriasis tagSNPs in functional genomics data of psoriasis relevant cells, we implemented an enrichment test through variant set enrichment (VSE) R package [28]. We identified 26 types of human primary cells/cell lines, mainly primary immune cells and skin keratinocytes cells, in NIH Roadmap Epigenomics Consortium. They have been widely suggested to be relevant to the pathophysiology of psoriasis. We then downloaded the consolidated narrow peaks data of five core histone marks for these cells (i.e. H3K27ac, H3K27me3, H3K36me3, H3K4me1, and H3K4me3, S2 Table)[29]. The quality and peak calling have been described in detail in previous studies and on website http://egg2.wustl.edu/roadmap/web_portal/processed_data.html#ChipSeq_DNaseSeq [30]. An enrichment analysis was also conducted through R VSE package in ChIP-seq data for 111 transcription factors (TFs). We downloaded the narrow peaks data for TFs within GM12878 lymphoblastoid cell line from ENCODE consortium [24]. The peaks were quantified by conservative irreproducible discovery rate (IDR).

The VSE R package computes whether psoriasis associated variant set (AVS), i.e. a set of psoriasis tagSNPs, is significantly enriched in tested genomic feature, i.e. histone marks and TFs. For each of the 75 tagSNPs, we obtained its respective LD block, and all correlated SNPs that were in high LD (r^2^ ≥ 0.8 in 1000 Genomes Project EUR or EAS populations). In order to compute the enrichment score and its respective *p* value, we built null matched random variant sets (MRVSs) of size 1,000. The MRVSs are identical to AVS block by block on minor allele frequency (MAF), proximity to transcription start sites (TSS), and LD structure. All of these analyses were accomplished in R 3.4.2. A *p* value < 0.05 was used as significant threshold after Bonferroni correction of number of histone marks.

### Prioritization of functional SNPs

We searched exhaustively for all highly correlated SNPs (LD r^2^ ≥ 0.8) (termed as “correlated SNPs”) residing in a 1Mb-window centered on the 75 tagSNPs in the 1000 Genomes Project Asian and European populations reference panels, respectively (May 2012 data release, http://www.1000genomes.org/). We downloaded five core chromatin state files (i.e. H3K27ac, H3K27me3, H3K36me3, H3K4me1, and H3K4me3) for CD4 naïve primary peripheral blood T cells from the NIH RoadMap Epigenomic Consortium (http://www.ncbi.nlm.nih.gov/geo/roadmap/epigenomics/). Increased frequencies of CD4+ T cells have been consistently discovered in psoriatic skin lesions [31]. Besides, a role of Th1 CD4 T cells was initially suspected and Th17 CD4 T cells have been shown to play a major role in psoriasis recently [32]. We queried the biological function for each correlated SNP in functional genomic data sets. The analyses were accomplished in R/Bioconductor FunciSNP package [33].

We categorized the correlated SNPs into five groups by the priority of exonic variants, gene promoters, enhancers, transcribed, and repressed regions. Coding exon data was downloaded from the UCSC genome table browser [34]. The gene promoters, enhancers, transcribed, and repressed regions, were identified by the overlaps of histone modifications H3K4me3, H3K27ac or H3K4me1, H3K36me3, and H3K27me3, respectively. Gene promoter variants were limited within -1000 and +100 base pairs from TSS. All statistical tests were implemented in R 3.4.2.

### Variants annotation

We used bioinformatics tools to characterize the correlated SNPs’ functional potentials, e.g. consequence on protein structures for coding variants and perturbation of TF binding affinity for non-coding variants. The functional consequences for exonic variants were analyzed through PolyPhen2 and SIFT using default parameters [35, 36]. For the non-coding genome sequences, they might contribute to the risk of psoriasis through regulating gene expression via various mechanisms, for example affecting TF binding affinity. We searched for the TF motifs binding effect to each of the correlated SNPs using motifbreakR R package [37].

## Results

### Enrichment of histone marks and transcription factors in psoriasis

**Fig 1:**
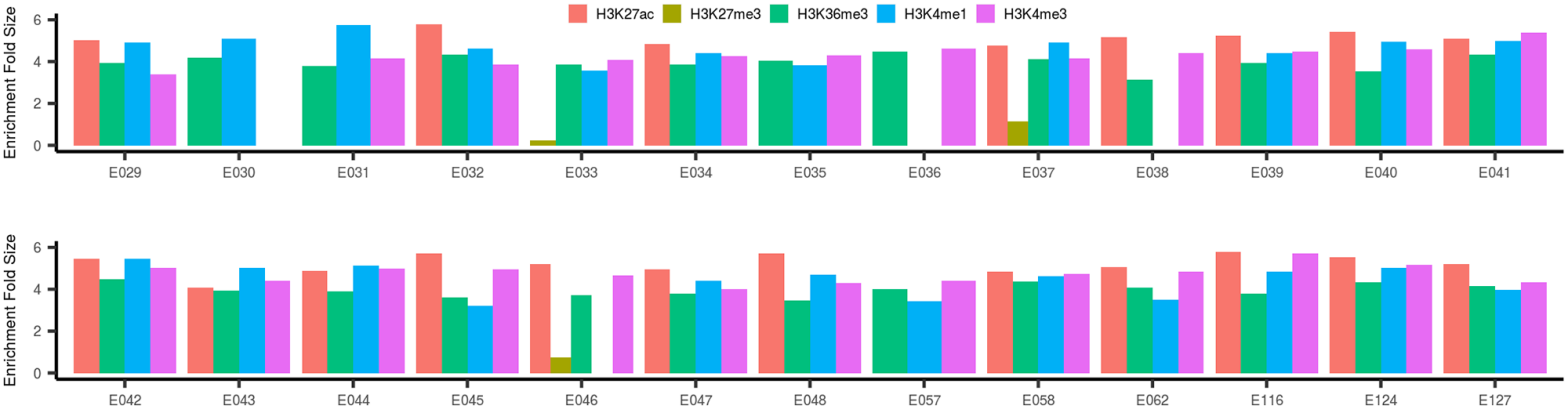
The enrichment pattern of five core histone markers in 26 relevant tissues/cells for psoriasis. E029:CD14_Primary_Cells; E030:CD15_Primary_Cells; E031:CD19_Primary_Cells_Cord_BI; E032:CD19_Primary_Cells_Peripheral_UW; E033:CD3_Primary_Cells_Cord_BI; E034:CD3_Primary_Cells_Peripheral_UW; E035:CD34_Primary_Cells; E036:CD34_Cultured_Cells; E037:CD4_Memory_Primary_Cells; E038:CD4_Naive_Primary_Cells; E039:CD4+_CD25-_CD45RA+_Naive_Primary_Cells; E040:CD4+_CD25-_CD45RO+_Memory_Primary_Cells; E041:CD4+_CD25-_IL17-_PMA-Ionomycin_stimulated_MACS_purified_Th_Primary_Cells; E042:CD4+_CD25-_IL17+_PMA-Ionomcyin_stimulated_Th17_Primary_Cells; E043:CD4+_CD25-_Th_Primary_Cells; E044:CD4+_CD25+_CD127-_Treg_Primary_Cells; E045:CD4+_CD25int_CD127+_Tmem_Primary_Cells; E046:CD56_Primary_Cells; E047:CD8_Naive_Primary_Cells; E048:CD8_Memory_Primary_Cells; E057:Penis_Foreskin_Keratinocyte_Primary_Cells_skin02; E058:Penis_Foreskin_Keratinocyte_Primary_Cells_skin03; E062:Peripheral_Blood_Mononuclear_Primary_Cells; E116:GM12878_Lymphoblastoid; E124:Monocytes-CD14+_RO01746; E127:NHEK-Epidermal_Keratinocytes.

**Fig 2:**
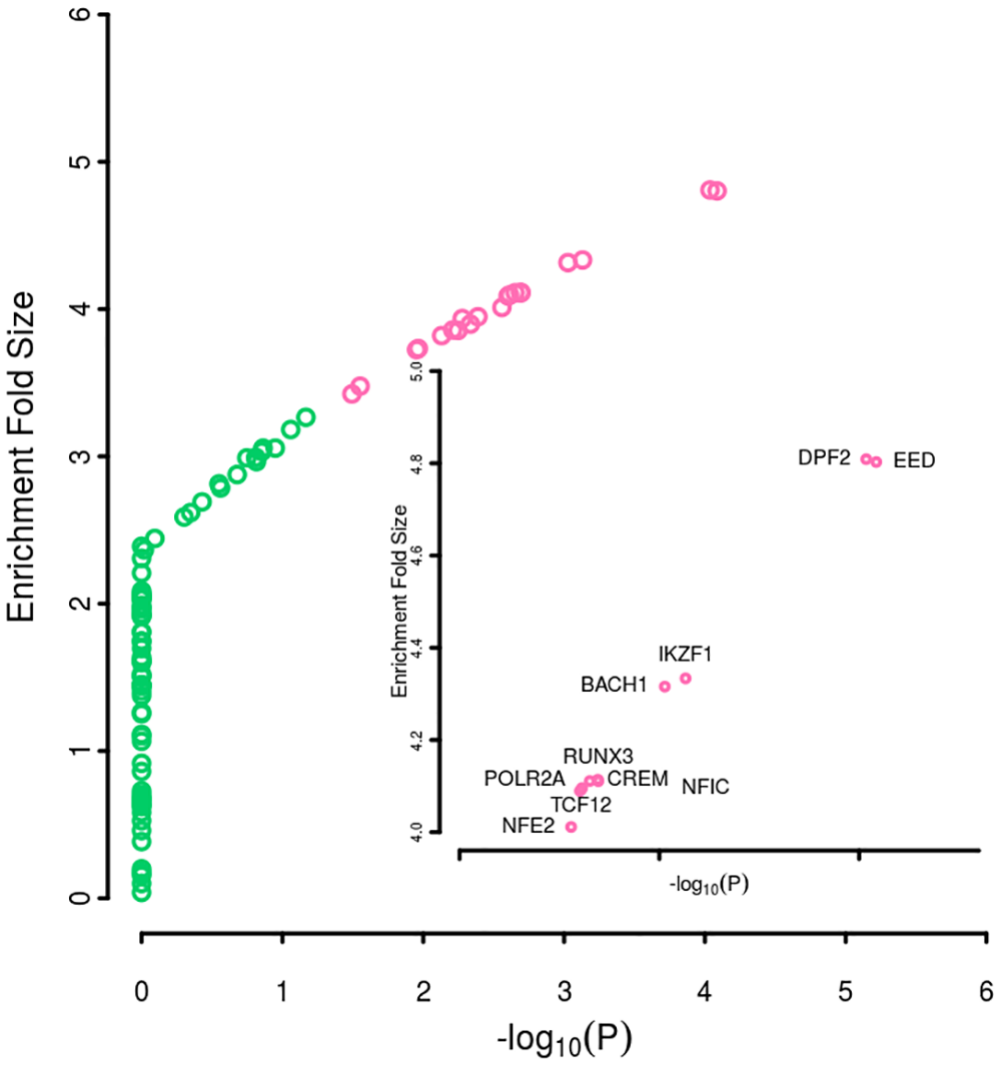
The enrichment of transcription factors within psoriasis association regions. The x-axis denotes the -log transformed p values in the transcription factors enrichment test. The y-axis represents the enrichment fold size when comparing with the null distribution. Each circle in the plot denotes a transcript factor. The top transcription factors with enrichment p value < 0.05 are colored in pink. The embedded plot shows the names the names of transcription factors with enrichment fold size over 4.

Aberrant immune activities have been implicated widely in the pathophysiology of psoriasis. Genetic variants might affect the risk of psoriasis through regulating the immune levels. To characterize the enrichment pattern of psoriasis tagSNPs across functional genomic biofeatures in various immune cells, we evaluated the enrichment of 75 psoriasis tagSNPs against five core histone marks in each of 26 relevant tissue/cell. We found that psoriasis genetic variants were pervasively significantly enriched in the five core histone marks in 26 relevant cells and cell lines (Fig 1). We showed the enrichment pattern in each type of cells was almost consistent especially for histone marks H3K27ac, H3K4me1, and H3Kme3 though a difference existed (Fig 1). Among the 111 TFs in human blood lymphoblastoid cell lines, we identified 17 TFs, for example *EED* (enrichment fold = 4.80, *p* = 8.20E-5), *RUNX3* (enrichment fold = 4.11, *p* = 2.03E-3), *IKZF1* (enrichment fold = 4.33, *p* = 7.39E-4), and *TCF12* (enrichment fold = 4.09, *p* = 2.50E-3) that significantly bind with psoriasis genetic variants (Fig 2 and S3 Table).

### Psoriasis potential functional variants

**Fig 3:**
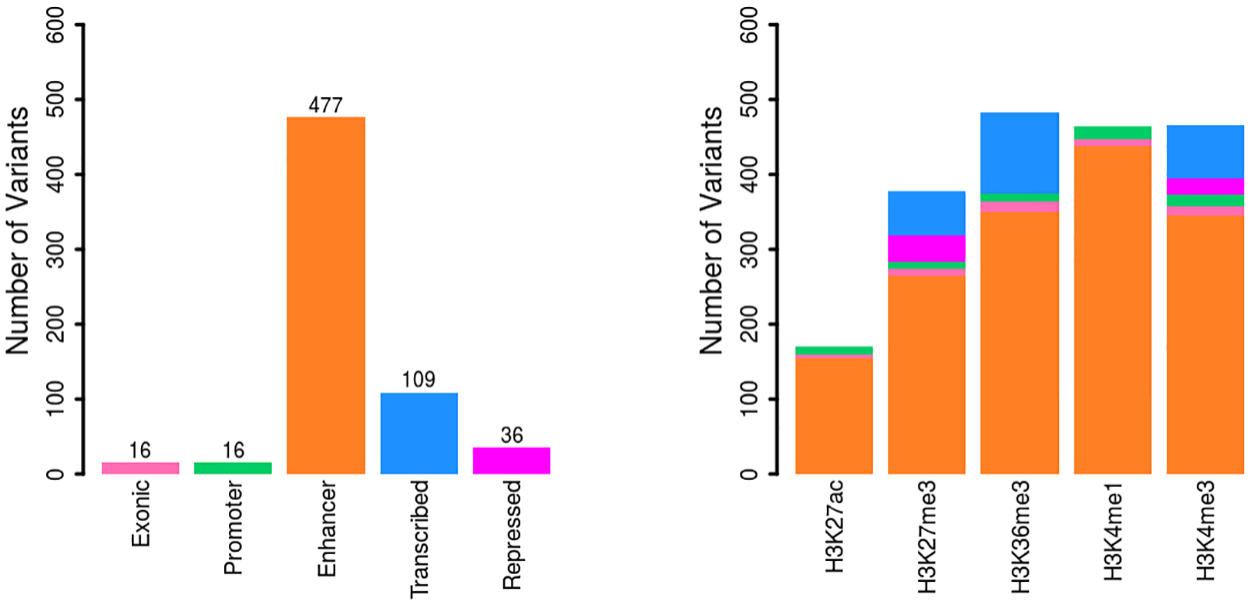
The category and summary statistics of 654 potential putative functional variants for psoriasis in CD4+ primary T cells.

Within psoriasis susceptibility regions, there were totally 1,609 correlated SNPs strongly linking with the 75 unique psoriasis tagSNPs (LD r^2^ ≥ 0.8). We identified 654 SNPs of them overlapped with at least one of the five core histone marks in CD4+ primary T cells (S4 Table). Given the key pathogenic role of CD4+ primary T cells in psoriasis pathophysiology, these 654 SNPs are considered as PFV within psoriasis GWAS regions. In order to verify the functional potential of PFV set, we tested the overlapping between these 654 SNPs and the previously implicated causal variants for autoimmune diseases [38]. Among the 654 PFVs, there were 148 SNPs overlapping with the autoimmune diseases causal variants. The proportion of overlapping was statistically significantly higher than that among the background variants (N = 973) that are strongly linked with the 75 unique tagSNPs but non-PFV (Z statistics = 14.47, *p* = 1.43E-4). Of these 654 potential functional SNPs, we identified 16 exonic sequences. In addition, we found 16, 477, 109, and 36 SNPs of them within gene putative promoters, enhancers, transcribed, and repressed regions, respectively (Fig 3).

### Annotation of potential putative functional variants

**Fig 4:**
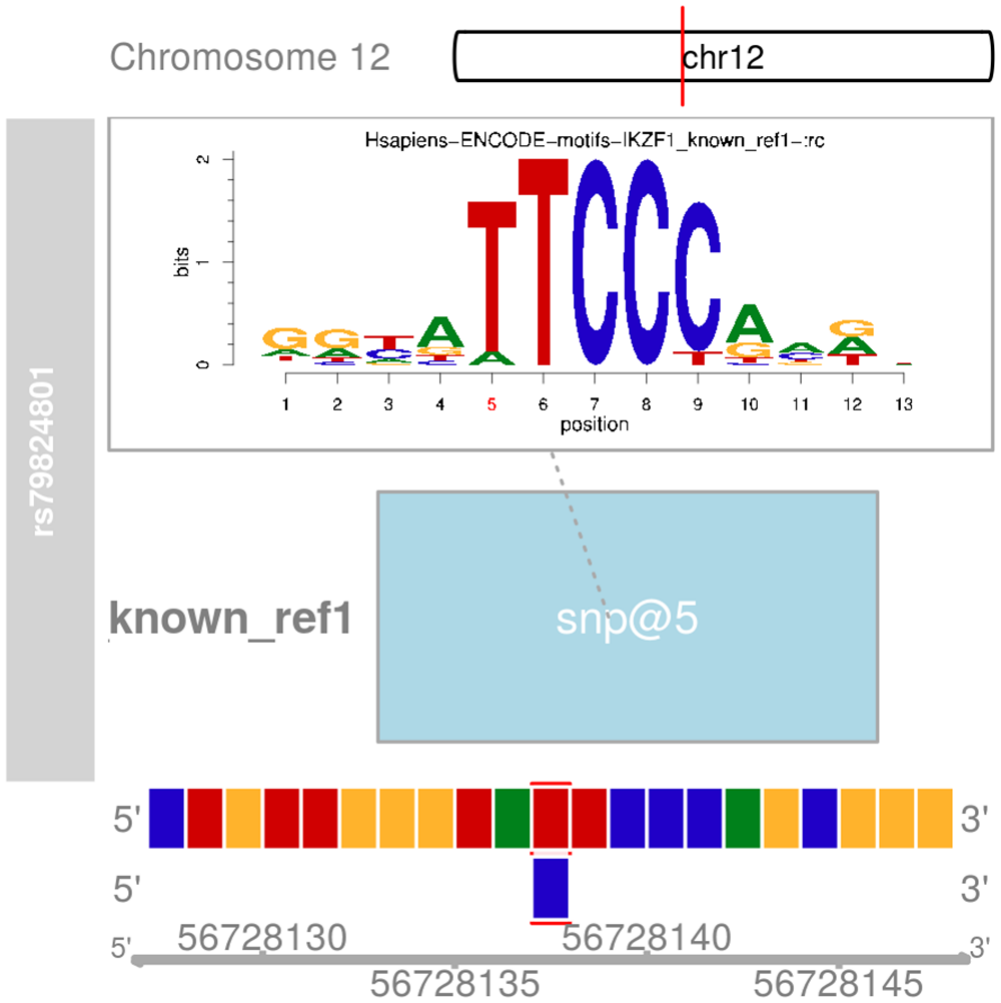
The binding effect of transcription factor *IKZF1* with potential putative functional variant rs79824801. Genomic coordinates are shown in the bottom panel. The position of rs79824801 is indicated with red and blue bounding box for reference and alternative alleles, respectively. The position of rs79824801 is represented in light blue box in the middle panel., and. The top panel shows the motif logos generated from motifstack, with the position of rs79824801 labeled in red.

Of the 16 exonic variants, we identified a missense variant SNP rs27044 in exon of 15 in the gene *ERAP1*. In addition, the majority of 654 potential putative functional variants were non-coding sequences suggesting they might contribute to the risk of psoriasis through regulating gene expression via various mechanisms, for example affecting TF binding affinity. We studied further SNP rs79824801 in the PFV set as an example. SNP rs79824801 is strongly linked with psoriasis tagSNP rs2066819 (LD r^**2**^ > 0.9 in both European and Eastern Asian populations in 1000 Genomes Project) on chromosome 12. It was simultaneously located in peaks of multiple histone marks, i.e. H3K27me3, H3K27ac, H3K4me1, H3K3me3, and H3K36me3 in CD4+ primary T cells, suggesting its potential functional role in CD4+ primary T cells. We found that its sequence changed the binding affinity of TF *IKZF1* that was suggested with significant enrichment (Fig 4). The binding effect with the alternative allele C was significantly lower.

## Discussion

In the present study, we implemented an integrative analysis to interrogate each highly linked variant of 75 unique psoriasis GWAS findings and to prioritize potential putative functional variants from background genetic variants within psoriasis GWAS association regions. We established eventually a catalog of 654 potential putative functional variants and suggested that they were significantly enriched among the causal variants for autoimmune diseases. We characterized a pervasive enrichment pattern of psoriasis GWAS findings in five core histone marks across various psoriasis relevant cells/tissues. We discovered 17 TFs significantly binding with psoriasis GWAS regions. This study highlights the complex genetic architecture and biological mechanisms for psoriasis. The findings would inform the functional experiment design for psoriasis.

In order to investigate the functional role of genetic variants which have been identified in GWASs, to use the most relevant cells is pivotal. It is challenging to prioritize the relevant cells for human complex diseases. It has been suggested that the integrative analyses with publicly available functional genomic data will help narrow down the spectrum of effector cells [21, 27]. Aberrant immune activities have been widely identified in the pathogenesis of psoriasis. However, the main effector immune cells are still unclear. We integrated psoriasis GWASs association variants with epigenomic data from a wide spectrum of immune tissues/cells. We prioritized a number of immune cells in the pathogenesis of psoriasis, and identified a pervasive enrichment pattern of psoriasis variants within epigenetic histone marks from immune tissues/cells. This study suggests that it is challenging to identify the effector immune cells, and therefore choose the most appropriate cells to study the biological consequences of psoriasis causal variants in the post GWAS era. These findings highlight a complex disease mechanism of psoriasis.

In the present study, we integrated psoriasis GWAS association variants with publicly available functional genomic data in CD4+ primary T cells, and achieved a relatively comprehensive catalog of PFVs in psoriasis GWAS regions. Of them, SNP rs27044 is predicted to be a missense variant in the gene *ERAP1*, which results in an amino acid change of glutamic acid from glutamine in the protein product, then supporting its causal role of *ERAP1* gene in this region. SNP rs27044 has been prioritized to be a causal variant for autoimmune diseases [38], and interacts with HLA-C*06 in the development of early onset psoriasis patients [39]. Recent studies have shown a CC haplotype of SNPs rs30187/rs27044 within the gene *ERAP1* confers a protection effect to psoriatic arthritis (PsA) [40]. Though further experiments are warranted, we suggest SNP rs27044 as a causal variant in the gene *ERAP1* for psoriasis.

We discovered in primary CD4+ T cells that the majority of psoriasis PFVs are resided in non-coding genome regions, especially in gene enhancer elements. This finding is consistent with previous discoveries that a dominant overlap of human complex disease GWAS findings with genetic variants within gene enhancers [41]. Given key role of the non-coding variants in the risk of human complex diseases, they may contribute through regulating gene expression level rather than altering protein structure [42]. However, the affected mechanisms of gene expression levels are complex and largely remain unclear. A well-known common mechanism is through binding a specific TF [43]. We conducted a TF enrichment analysis in psoriasis GWAS variants, and screened 17 TFs significantly binding to the psoriasis variants. Some of these top TFs play a role in the processes of cytokine regulation, and cell differentiation and proliferation, which are shown to be important in the etiology of psoriasis [44]. For example, runt-related transcription factor 3 (*RUNX3*) has been reported to be a susceptibility gene for psoriasis and promotes Th1 cells differentiation through binding with T-bet [5, 45]. A recent study found that increased miR-138 regulates the balance of Th1/Th2 through inhibiting *RUNX3* expression in CD4+ T cells in psoriasis [46]. The inhibition of *RUNX3* reduced the relevant cytokines levels, and decreased the frequency of Th17 and Th22 cells in CD4+ T cells from psoriasis patients [47]. Taken together, these results suggest that *RUNX3* may act a promising therapeutic target for psoriasis. We prioritized SNP rs79824801 on chromosome 12 as a PFV in *IL23A* region for psoriasis. This SNP resides in multiple histone peaks in CD4+ primary T cells. Its alternative allele would change the binding effect of TF *IKZF1* that was suggested in our TF enrichment analysis in this study. *IKZF1* played multiple important roles on regulators of lymphocyte differentiation [48]. Though the evidence between *IKZF1* and the risk of psoriasis is limited, several studies have demonstrated its relationship with autoimmune disease systemic lupus erythematosus (SLE) [49]. *IKZF1* has been suggested to affect the STAT4 and IFN pathways [50–52], both of which involve in the pathogenesis of psoriasis [53–55]. Further studies are worth to validate rs79824801 in an independent sample, to quantify the binding affinity of *IKZF1* at this site, and to evaluate the consequence of IFN signaling in the biological mechanism of psoriasis.

There are several limitations. Firstly, we only evaluated the roles of common variants. Secondly, the functional genomic data was not extremely comprehensive regarding its sequencing depth and study sample size. We did not correct for the number of studied tissues/cells in the enrichment analyses. Finally, we only used epigenetic profiles from relevant normal tissues/cells. The epigenetic data from disease tissues/cells is lacking.

In summary, we characterized a complex genetic architecture of psoriasis, and established a relatively comprehensive catalog of common potential putative functional variants for psoriasis. The findings would guide the functional experiments for psoriasis in future.

## Supporting information

S1 Table The 75 TagSNPs with genome-wide significance for psoriasis. (XLSX)

S2 Table The enrichment of histone marks in 26 relevant tissues/cells for psoriasis. (XLSX)

S3 Table The enrichment of 111 transcription factors within psoriasis association regions. (XLSX)

S4 Table The 654 potential putative functional variants within psoriasis GWAS regions. (XLSX)
