# Correction: A catalog of potential putative functional variants in psoriasis genome-wide association regions

**DOI:** 10.1371/journal.pone.0199322

**Published:** 2018-06-14

**Authors:** Yan Lin, Lu Liu, Yujun Sheng, Changbing Shen, Xiaodong Zheng, Fusheng Zhou, Sen Yang, Xianyong Yin, Xuejun Zhang

The following information is missing from the Funding section: This study was supported by National Natural Science Foundation of China grant 81130031 to Xuejun Zhang and National Natural Science Foundation of China grants 81370044 and 81000692 to Xianyong Yin. This study was also supported by Excellent Youth Foundation of Anhui Scientific Committee grant 1808085J08 to Xianyong Yin.

## References

[1] Lin Y, Liu L, Sheng Y, Shen C, Zheng X, Zhou F, et al (2018) A catalog of potential putative functional variants in psoriasis genome-wide association regions. PLoS ONE 13(5): e0196635 https://doi.org/10.1371/journal.pone.0196635 29715312 10.1371/journal.pone.0196635 PMC5929547

